# OHVIRA Syndrome and Ureteral Ectopy Draining in the Ipsilateral Hemiuterus, Diagnosed in the Prepubertal Age Group: Case-Report and Literature Review

**DOI:** 10.3390/medicina60121922

**Published:** 2024-11-22

**Authors:** Teodora Telecan, Roxana Denisa Capras, Gabriela Adriana Filip, Emanuela Maria Ionutas, Dan Vasile Stanca, Carmen-Bianca Crivii

**Affiliations:** 1 Department of Anatomy and Embryology, “Iuliu Hatieganu” University of Medicine and Pharmacy, 400012 Cluj-Napoca, Romania; t.telecan@gmail.com (T.T.); gabriela.filip@umfcluj.ro (G.A.F.); bianca.crivii@umfcluj.ro (C.-B.C.); 2 Department of Pathology, County Emergency Clinical Hospital, 400347 Cluj-Napoca, Romania; 3 Department of Urology, “Iuliu Hatieganu” University of Medicine and Pharmacy, 400012 Cluj-Napoca, Romania; emanuelaionutas@gmail.com (E.M.I.); vasilestanca@umfcluj.ro (D.V.S.); 4 Department of Urology, Clinical Municipal Hospital, 400139 Cluj-Napoca, Romania

**Keywords:** OHVIRA syndrome, ectopic ureter, uterus, prepubertal, 3D laparoscopic nephrectomy

## Abstract

*Background:* Müllerian (paramesonephric) duct anomalies (MDA) are a rare condition, occurring in 5.5% of female newborns. One of the most complex malformations is represented by Obstructed Hemivagina and Ipsilateral Renal Anomalies (OHVIRA) syndrome, also known as Herlyn –Werner–Wunderlich (HWW) syndrome. *Case presentation:* We present the case of a 7-year-old asymptomatic premenarchal female patient diagnosed with OHVIRA syndrome with ipsilateral renal hypoplasia and ectopic ureteral implantation at the level of the uterus. As the patient developed urinary incontinence after the incision of the vaginal septum, right-sided 3D laparoscopic total nephrectomy was performed. *Literature review:* OHVIRA syndrome associated with ureteral ectopy is a rare occurrence, being encountered in 0.0064% of cases. The premenarchal diagnosis represents a challenge, due to the underdeveloped status of the genital tract. However, it should be ruled out in female newborns with unilateral renal agenesia or multicystic dysplastic kidney. Most reported cases describe the obstructed hemivagina as the site of ureteral ectopy. To our knowledge, this is the first reported case of OHVIRA syndrome with ectopic ureter draining at the level of the ipsilateral hemiuterus, diagnosed before the pubertal age. *Conclusions:* OHVIRA syndrome is one of the rarest Müllerian duct abnormalities. The management of these patients should be conducted in multidisciplinary teams, with long-term urological and gynecological follow-ups.

## 1. Background

Müllerian (paramesonephric) duct anomalies (MDA) are a rare condition, occurring in 5.5% of female newborns and affecting the development of the upper two-thirds of the vagina, cervix, corpus uteri, and Fallopian tube [1]. One of the most complex malformations is represented by Obstructed Hemivagina and Ipsilateral Renal Anomalies (OHVIRA) syndrome, also known as Herlyn–Werner–Wunderlich (HWW) syndrome. It is characterized by a didelphic uterus with imperforated hemivagina, associated with ipsilateral renal defects such as agenesia, hypoplasia or ectopic ureter [2]. It represents 0.1% to 3.8% of all diagnosed MDA [3].

Ureteral ectopia is defined as the abnormal insertion of the ureter, beyond the bladder trigone. It is attributed to the failure of the ureteral bud to detach from the Wolffian (mesonephric) duct, a condition favored by concomitant MDA [4]. In female patients, the ureteral orifice is most frequently found at the level of the urethra (45%), vagina (35%), or vulval vestibule (15%), while the uterus and the Fallopian tubes are one the rarest locations (under 5%) [5].

In female patients, ureteral ectopia leads to urinary incontinence, as the implantation point is always below the urinary sphincter. Therefore, the patients tend to become symptomatic in their early childhood [6]. However, OHVIRA syndrome is diagnosed during puberty, when dysmenorrhea and abdominal pain are present due to the forming hematocolpos. Diagnosis at prepubertal age is a rare occurrence, most frequently being made incidentally, during routine evaluation of the already known renal malformation [7].

We present the case of a premenarchal female patient diagnosed with OHVIRA syndrome with ipsilateral renal hypoplasia and ectopic ureteral implantation at the level of the uterus, who underwent right total nephrectomy as treatment for urinary incontinence.

## 2. Case Presentation

We present the case of a 7-year-old asymptomatic female patient, who was addressed to our department for suspicion of complex urogenital malformation. Her past medical history revealed at 2 months of age the absence of a visible, normally developed right renal unit, with 2–3 cystic masses identified in the right ilio-lumbar area. Otherwise, the abdomen was not distended nor with other notable changes, the patient being asymptomatic. At that point, the case was interpreted as unilateral complete cystic degeneration of the right kidney. Due to the asymptomatic clinical presentation and normal routine bloodwork, a conservative attitude was adopted. The patient followed periodic evaluation every three months, abdominal ultrasound and serum creatinine being within normal limits, with the initially discovered transonic images remaining stationary in size.

**Figure 1:**
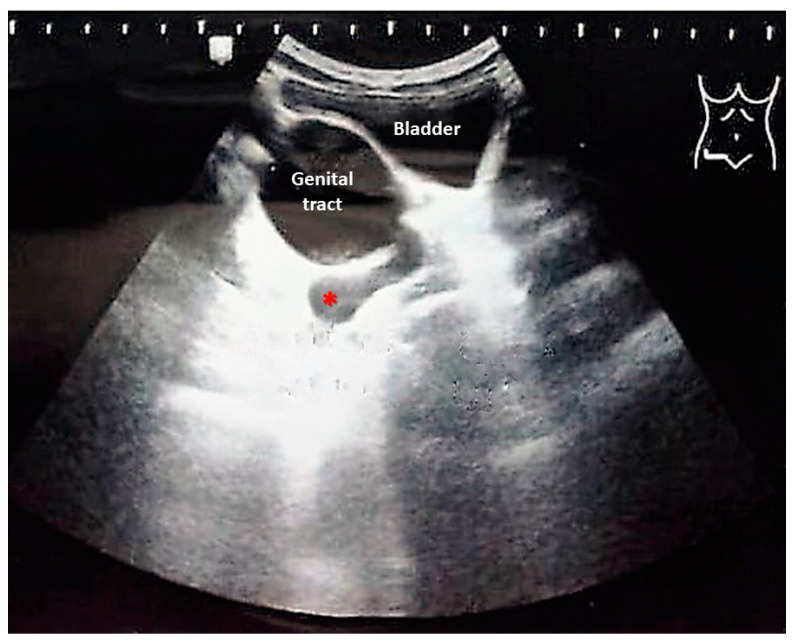
Abdominal ultrasound showing the genital tract filled with fluid, as well as hydrosalpinx (red asterisk).

In March of 2022, the routine ultrasound revealed a mass of approximately 30 mm with thin walls and transonic content, in the right paramedian and posterior plane in relation to the bladder (Figure 1).

**Figure 2:**
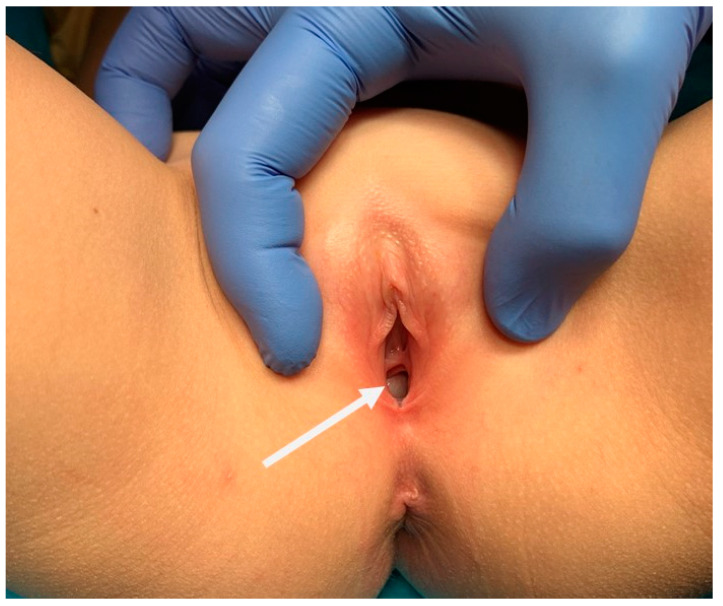
Normally developed vaginal introit and urethral orifice. Transverse septum protruding posteriorly to the hymenal fold (white arrow).

Upon physical examination of the urogenital apparatus, the urethral orifice and vaginal introit appeared to be normally developed. A transversal vaginal septum protruded posteriorly to the hymenal fold, occupying the right half of the vaginal canal (Figure 2).

**Figure 3:**
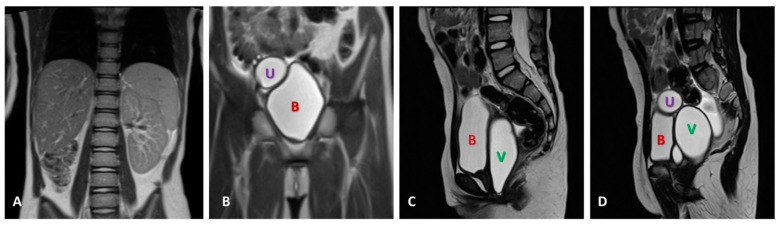
MRI abdominal and pelvic images. (**A**). Absence of the right kidney, with contralateral hypertrophic kidney (frontal view). (**B**). Distended urinary bladder (B) and right uterine cavity (U) filled with fluid (frontal view). (**C**). Distended right hemivagina (V), visible behind the bladder (sagittal view). (**D**). Hydrometrocolpos (sagittal view).

The imagistic diagnosis was further completed with contrast-enhanced abdominal and pelvic magnetic resonance imaging (MRI), that highlighted at the level of the fourth lumbar vertebra, a reniform structure, measuring 20 mm in the cranio-caudal axis and being interpreted as a hypoplastic ectopic kidney. The contralateral renal unit presented compensatory hypertrophy (97 × 47 × 42 mm), with preserved corticomedullary differentiation. The right ureter could be visualized entirely, measuring 5 mm at the maximum diameter. Additionally, the uterus was found to have a which had a didelphic conformation, The opening of the ureter was assessed to be at the level of the right-sided hemiuterus, in the outer third of the fundus uteri. The right adnexa, uterus and vagina were distended and filled with fluid content (Figure 3), measuring 33 mm in the latero-lateral axis. Thus, the initial ultrasonographic diagnosis of unilateral complete cystic degeneration of the right kidney was refined, the diagnosis of hypoplastic right kidney and associated right hydrosalpingometrocolpos being established.

**Figure 4:**
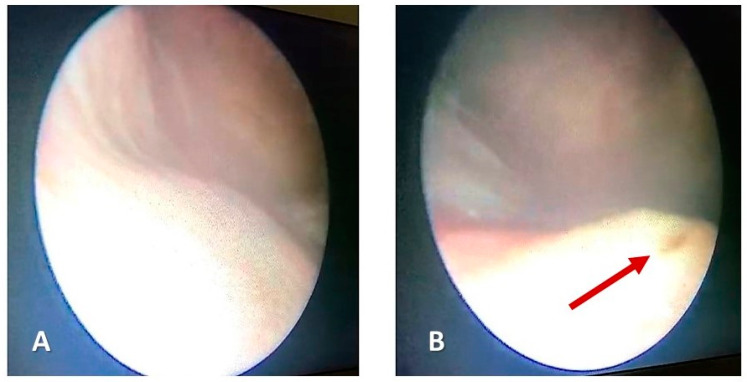
Retrograde urethrocystoscopy. (**A**). Absence of the right ureteral orifice. (**B**). Normally conformed ureteral orifice on the left side (red arrow).

After obtaining the written informed consent of the legal representative, a retrograde urethrocystoscopy was performed under general anesthesia, which objectified the absence of the right ureteral opening at the level of the trigone, with normally developed ureteral orifice on the left side (Figure 4).

The vaginal septum was incised by the gynecologist as the second part of the evaluation, with approximately 200 mL of serous fluid being evacuated and sent for further analysis. The creatinine level of the retrieved fluid sample was elevated, measuring 50.03 mg/dL (compared to the patient’s serum creatinine of 0.5 mg/dL), thus confirming that the drained fluid was urine.

The patient was reevaluated 1 month after the procedure. The abdominal ultrasound showed no sign of fluid accumulation within the genital tract. However, the patient developed urinary incontinence, with few milliliters of urine being voided transvaginally throughout the day. At this point, due to the newly developed urinary incontinence, total nephrectomy of the ectopic kidney was proposed.

**Figure 5:**
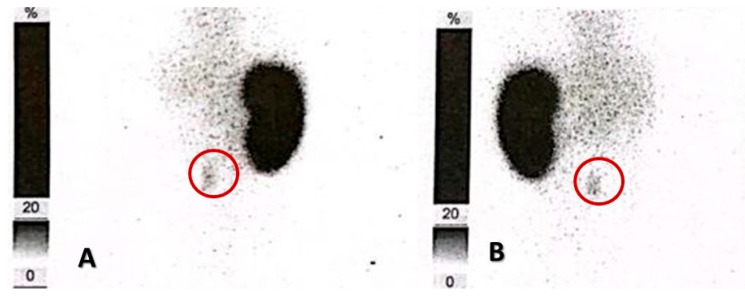
Tc-99m-DMSA renal scintigraphy, showing an enlarged, hyperfunctioning left kidney, as well as a hypoplastic right kidney (red circle), with little function preserved. (**A**). Anterior view. (**B**). Posterior view.

Prior to intervention, the patient underwent static renal scintigraphy with Technetium-99m-labeled dimercaptosuccinic acid (Tc-99m-DMSA), that showed a differential renal function of 99% of the left kidney and 1% for the right one (Figure 5), thus further confirming the presence of a hypoplastic right kidney. From a functional point of view, the indication of right-sided total nephrectomy was justified, as it would have little to no impact on the overall renal function and creatinine clearance.

We performed 3D laparoscopic transperitoneal right total nephrectomy, using Aesculap system (B. Braun, Tuttlingen, Germany). The patient was placed in left lateral decubitus, with the surgical table bent at a 45 degree angle, to better expose the lumbar area. Three working trocars were placed in a triangular fashion, as follows: a 10 mm optic trocar was placed 3–4 cm paraumbilically, at the lateral margin of the rectus abdominis muscle. The 3 mm working trocar for the left hand was placed in the right inguinal fossa, medially to the right antero-superior iliac spine. The 10 mm working trocar for the right hand was inserted 2 cm below the costal margin, on the anterior axillary line.

**Figure 6:**
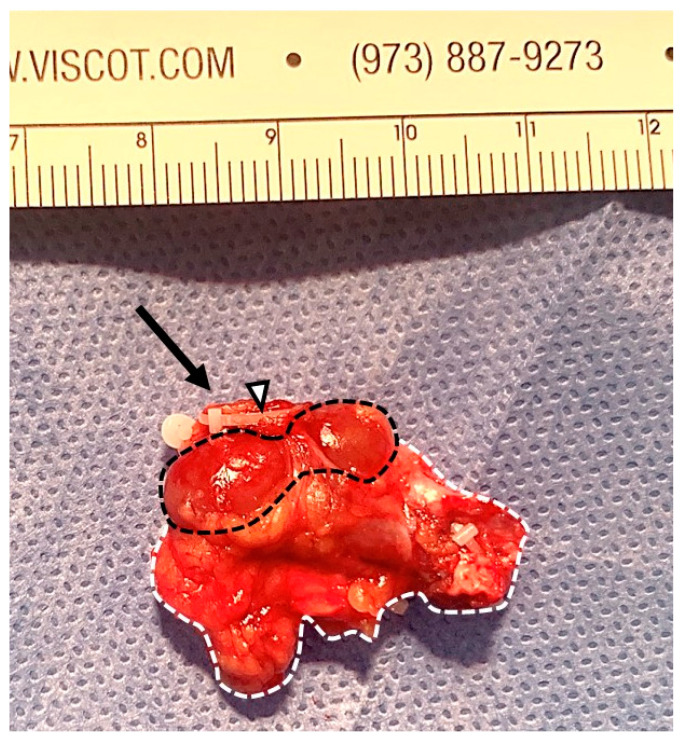
Surgical specimen measuring 35 × 20 mm; right hypoplastic kidney, measuring 20 × 10 × 5 mm (Dotted black line = the outline of the kidney; dotted white line = the outline of the adjacent, perirenal adipose tissue; black arrow = renal pedicle; white arrowhead = non-resorbable vascular sealing clip).

After inspecting the abdominal cavity, the parietal peritoneum was incised laterally to the cecum and medialized. Upon inspection, the Müllerian duct anomalies described on MRI were confirmed, the uterus having a complete didelphic conformation, with only one vaginal fornix being identified. Due to its ectopic position, the right ureter could be visualized as a structure descending towards the right corpus uteri. The absence of pyelocaliceal or ureteral duplication was noted. The right kidney was identified anterolaterally to the inferior vena cava, above the confluence of the common iliac veins. The renal pedicle was composed of one artery emerging from the aorta and two renal veins, that were draining into the right ovarian vein. Each element was sealed using Hem-o-Lok clips (Teleflex Surgical, Morrisville, North Carolina, USA) and divided. After circumferential dissection of the right renal unit, the surgical specimen was removed in an endobag (Figure 6). A peritoneal drainage tube was placed at the level of the right iliac fossa and the access points were sutured.

The total intubation time was 148 min. The patient’s recovery was uneventful. The bladder catheter and drainage tube were removed on the second postoperative day (POD) and the patient was discharged on the fourth POD.

The pathological report described the kidney as having corticomedullary differentiation, with four renal pyramids and narrow renal pelvis. Microscopically, no signs of atrophy, cystic degeneration or dysplasia were observed.

At the 2-month postoperative follow-up, the patient remained asymptomatic, with surgical wounds healed per primam. The postoperative creatinine level was 0.56 mg/dL. She and her relative described the absence of urinary incontinence or vaginal secretions after the surgery. The abdominal ultrasound did not reveal de novo fluid build-up within the genital tract.

The patient was scheduled to subsequent periodical follow-ups, every 3 months in the first postoperative year and thereafter biannually, until puberty. Long-term, yearly follow-up was advised, as the full impact of the MDA on the patient’s fertility could not be assessed at this stage. Up to the publication date of the current case report, the patient was followed for a period of 18 months. The serum creatinine lever remained within physiological range. The abdominal ultrasounds were negative for newly formed collections at the level of the right-sided salpinx, hemiuterus and hemivagina.

## 3. Literature Review

OHVIRA syndrome was initially described as the Müllerian duct anomaly associated exclusively with ipsilateral renal agenesis [8,9]. However, the prevalence of OHVIRA case reports associating ipsilateral renal anomalies has increased in the past decade, with Schlomer et al. [10] suggesting that the definition should accept the boarder term of ‘renal anomaly’ rather than ‘agenesis’.

In female patients, 80% of ectopic ureters are linked to a duplex renal system, whereas 20% develop alongside a single system [6]. In the latter situation, the diagnosis is oftentimes hindered by the fact that the ureter drains a dysplastic or atrophic kidney, with minimal urinary output [11]. Ureteral ectopy is the rarest variant of Wolffian duct anomaly associated with OHVIRA syndrome, occurring in 0.0064% of diagnosed patients [12]. In cases where the ureteral opening is found at the level of the obstructed half of the genital tract, patients do not report urinary incontinence firsthand, only after the vaginal septum is incised [13].

Few cases of OHVIRA syndrome are diagnosed at prepubertal age, usually in relationship with the co-existing renal anomaly. In case of ectopic ureters, the chief complaint patients present with may be urinary incontinence or abdominal discomfort and vaginal mass protruding at the level of the introitus, if the ureteral opening is located in the occluded hemivagina [14]. A brief review of the published prepubertal OHVIRA cases with ureteral ectopy can be found in Table 1. Most cases had a single collecting system, while the most common site of ureteral ectopy being the ipsilateral imperforated vagina.

As some OHVIRA cases are associated with multicystic dysplastic kidneys (MCDK), the diagnosis can be set in the first weeks postpartum based on the absence of the renal unit in the corresponding lumbar fossa and associated cystic masses. In our case, although the suspicion of MDCK was raised when the patient was 2-months-old, further, more comprehensive imagistic investigations were carried out 7 years later, once the hydrosalpingometrocolpos was visible, thus refining the initial diagnosis: what was initially thought to be multiple renal cysts turned out to be a right-sided hydrosalpingometrocolpos in the context of the complex Müllerian duct malformation (OHVIRA) associating ureteral ectopy at the level of the obstructed hemiuterus, while the right renal unit was visualized as being hypoplastic, slightly ectopic and without identifiable cystic structures. Kim et al. [14] suggested that in the presence of MCDK in female newborns, OHVIRA should always be ruled out by repeating the ultrasonographic examination at 1, 6 and 12 months of age, followed by periodic gynecological evaluation after thelarche, but before the beginning of menarche.

In terms of ureteral ectopy, Fallopian tubes and uterus are among the rarest implantation sites. Table 2 summarizes the cases published to date. In the absence of the obstructed hemivagina, patients report urinary incontinence or persistent dribbling of urine since early childhood [19]. In all cases, the affected kidney presented an ipsilateral duplicated collecting system.

Regarding treatment options, the cited literature debates between the conservative and surgical approach. OHVIRA cases are most frequently treated conservatively, by increasing the vaginal septum. However, in cases associated with ureteral ectopy, this leads to urinary incontinence, as the ipsilateral kidney, although presenting with important hypoplasia and reduced renal function (approximately 1–2%), still produces a small quantity of urine that voids vaginally, thus affecting the quality of life of the patients. Therefore, total nephrectomy is advised. In our case, we performed the septal incision as part of the diagnostic workflow. After assessing the differential renal function and taking into consideration the newly developed incontinence, total right-sided nephrectomy was performed.

On the other hand, when referring to ureteral ectopy in the absence of an obstructed ipsilateral hemivagina, the involved renal unit has a preserved function, thus reconstructive procedures being needed. Ghosh et al. [19] and Jain et al. [20] managed the reported cases by performing laparoscopic end-to-side ureteroureterostomy into the normal ureter of the lower moiety. Particularly, in the presence of non-functioning upper renal pole and dilated corresponding collecting system, heminephrectomy of the upper moiety is a valid alternative [21].

To summarize, the rarity of the proposed case report resides in the association between a complex genito-urinary malformation diagnosed at the prepubertal age and an uncommon site of ureteral ectopy.

## 4. Conclusions

OHVIRA syndrome is one of the rarest Müllerian duct abnormalities, this entity being suspected in the presence of unilateral multicystic dysplastic kidney in female newborns. The management of these patients should be performed in multidisciplinary teams, with long-term urological and gynecological follow-ups.

## Figures and Tables

**Table 1:** Cases of prepubertal patients diagnosed with OHVIRA syndrome and ureteral ectopy published to date.

No.	Author, Year	Age (Years)	Clinical Presentation	Site of Ureteral Ectopy	Management	Pathological Report	Outcome, Follow-Up
1.	Garge et al., 2014 [12]	10	Abdominal pain, suprapubic palpable mass, dysuria	Obstructed left hemivagina	Cystoscopy, vaginal septoplasty, diagnostic laparoscopy with left nephroureterectomy	Left oligonephric renal remnant	No vaginal stenosis or hydrocolpos 2 months of follow-up
2.	Nakahara et al., 2020 [15]	6	Absence of the left kidney and “pelvic cyst” diagnosed prenatally, with gradual distention	Obstructed left hemivagina	Transvaginal hemivagina septum resection, laparoscopic left ureter ligation and transection	N/A	No symptoms 5 years of follow-up
		1	Hydrometro-colpos diagnosed at birth	Between the right hemiuterus and right obstructed hemivagina	Transvaginal fluid aspiration, to confirm the presence of urine, diagnostic laparoscopy with right nephroureterectomy	Right dysplastic kidney	Uneventful recovery 2 years of follow-up
3.	Nassar et al., 2021 [11]	5	Continent, lower abdominal pain	Obstructed left hemivagina	Cystoscopy, vaginoscopy, septum incision, laparoscopic left nephroureterectomy	Left dysplastic kidney	Uneventful recovery 4 years of follow-up
4.	Romanski et al., 2021 [16]	11	Left lower quadrant pain	Cervix	Cystoscopy, hysterogram, diagnostic laparoscopy with left nephroureterectomy and left hemihysterectomy	Left dysplastic kidney	Uneventful recovery 6 months of follow-up
5.	Nakamura et al., 2022 [17]	4	Incontinence OHVIRA diagnosed at birth, vaginal septum incision	Right hemivagina (2 orifices)	Cystoscopy hysterosalpingography Diagnostic laparoscopy and right nephroureterectomy	Right hypoplastic kidney with bifid ectopic ureter	Resolution of incontinence Periodic renal function follow-up
6.	Khanal et al., 2023 [18]	0 (At birth)	Pre-natal ultrasound evaluation	Obstructed right hemivagina	Pre- and post-natal ultrasounds Magnetic resonance imaging (to be scheduled)	N/A	No symptoms Normal renal fucntion and urniary output Elective surgery to be scheduled

**Table 2:** Cases of patients diagnosed with ectopic ureter draining into the uterus published to date.

No.	Author, Year	Age (Years)	Clinical Presentation	Anatomical Variants	Management	Outcome
1.	Ghosh et al., 2016 [19]	13	Persistent dribbling of urine per vaginally since childhood, use of diapers on a regular basis	Right-sided duplex moiety with one of the ureters opening at the level of the uterus	Cystoscopy, vaginoscopy, diagnostic laparoscopy with end-to-side ureteroureterostomy between the ectopic ureter and the normally conformed one	Uneventful recovery Resolution of incontinence
2.	Jain et al., 2019 [20]	13	Urinary incontinence, pooling of urine at the level of the vaginal introitus	Right-sided duplex moiety with one of the ureters draining in the uterus	Cystoscopy, vaginoscopy, laparoscopic end-to-side ureteroureterostomy into the normal ureter of the lower moiety	Resolution of incontinence 6 months of follow-up
3.	Abyaksa et al., 2021 [21]	19	Urinary incontinence requiring 4–5 pads/day, not associated with effort or urgency	Right-sided duplex moiety with one of the ureters opening in the uterotubar junction	Cystoscopy, vaginoscopy, voiding cystourethrography, right laparoscopic heminephrectomy of the upper moiety	Uneventful recovery Resolution of incontinence

## Data Availability

The original contributions presented in this study are included in the article. Further inquiries can be directed to the corresponding author.

## References

[1] Engku-Husna E.I., Nik-Ahmad-Zuky N.L., Muhammad-Nashriq K. (2020). Müllerian duct anomalies with term pregnancy: A case report. J. Med. Case Rep..

[2] Kueppers J., Wehrli L., Zundel S., Shavit S., Stahr N., Szavay P.O. (2021). OHVIRA-syndrome in a newborn. J. Pediatr. Surg. Case Rep..

[3] Burgis J. (2001). Obstructive Müllerian anomalies: Case report, diagnosis, and management. Am. J. Obstet. Gynecol..

[4] Houat A.P., Guimarães C.T.S., Takahashi M.S., Rodi G.P., Gasparetto T.P.D., Blasbalg R., Velloni F.G. (2021). Congenital Anomalies of the Upper Urinary Tract: A Comprehensive Review. Radiographics.

[5] Lee D.G., Baek M., Ju S.H., Jeong B.C., Han D.H. (2011). Laparoendoscopic single-site nephrectomy for single-system ectopic ureters with dysplastic kidneys in children: Early experience. J. Laparoendosc. Adv. Surg. Tech. A.

[6] Demir M., Çiftçi H., Kılıçarslan N., Gümüş K., Oğur M., Gülüm M., Yeni E. (2015). A case of an ectopic ureter with vaginal insertion diagnosed in adulthood. Turk. J. Urol..

[7] Han J.H., Lee Y.S., Im Y.J., Kim S.W., Lee M.J., Han S.W. (2016). Clinical Implications of Obstructed Hemivagina and Ipsilateral Renal Anomaly (OHVIRA) Syndrome in the Prepubertal Age Group. PLoS ONE.

[8] Herlyn U., Werner H. (1971). Simultaneous occurrence of an open Gartner-duct cyst, a homolateral aplasia of the kidney and a double uterus as a typical syndrome of abnormalities. Geburtshilfe Frauenheilkd.

[9] Wunderlich M. (1976). Unusual form of genital malformation with aplasia of the right kidney. Zentralbl. Gynakol..

[10] Schlomer B., Rodriguez E., Baskin L. (2015). Obstructed hemivagina and ipsilateral renal agenesis (OHVIRA) syndrome should be redefined as ipsilateral renal anomalies: Cases of symptomatic atrophic and dysplastic kidney with ectopic ureter to obstructed hemivagina. J. Pediatr. Urol..

[11] Nassar H., Horst M., Gobet R. (2021). A rare case of symptomatic hydrometrocolpos in a 5y old female. Urol. Case Rep..

[12] Garge S., Bagga D., Acharya S.K., Yadav D.K., Khan T.R., Kumar R., Kumar V., Kumar S., Gupta D., Prasad A. (2014). Herlyn-Weber-Wunderlich syndrome with ectopic ureter in prepubertal female. J. Indian Assoc. Pediatr. Surg..

[13] Yang J.M., Yang S.H., Hsu H.C., Huang W.C. (2006). Transvaginal sonography in the morphological and functional assessment of segmental dilation of the distal ureter. Ultrasound Obstet. Gynecol..

[14] Kim Y.N., Han J.H., Lee Y.S., Lee I., Han S.W., Seo S.K., Yun B.H. (2021). Comparison between prepubertal and postpubertal patients with obstructed hemivagina and ipsilateral renal anomaly syndrome. J. Pediatr. Urol..

[15] Nakahara Y., Nakada S., Hitomi K., Hanaki S., Doi K., Goto T., Aoyama K. (2020). Urological anomalies associated with obstructed hemivagina and ipsilateral renal anomaly (OHVIRA) syndrome, a case series. J. Pediatr. Surg. Case Rep..

[16] Romanski P.A., Bortoletto P., Pfeifer S.M. (2021). Unilateral Obstructed Müllerian Anomalies: A Series of Unusual Variants of Known Anomalies. J. Pediatr. Adolesc. Gynecol..

[17] Nakamura M., Kanda S., Kajiho Y., Hinata M., Tomonaga K., Fujishiro J., Harita Y. (2023). A case of right hypodysplastic kidney and ectopic ureter associated with bicornuate uterus in a prepubertal girl. CEN Case Rep..

[18] Khanal L., Deora K., Gaihre S.R., Monika B. (2023). Obstructed Hemivagina and Ipsilateral Renal Agenesis Syndrome in a Neonate. Cureus.

[19] Ghosh B., Shridhar K., Pal D.K., Banerjee M. (2016). Ectopic ureter draining into the uterus. Urol. Ann..

[20] Jain P., Sarkar D., Maiti K., Gupta S., Pal D.K. (2019). Rare cases of ectopic ureter: Analysis from a single centre with review of the literature. Turk. J. Urol..

[21] Abyaksa R., Pramod S.V., Siregar S. (2021). Uterus as one of the ectopic ureter openings: Case report. Urol. Case Rep..

